# Indirect Calorimetry-Based Novel Approach for Evaluating Metabolic Flexibility and Its Association with Circulating Metabolic Markers in Middle-Aged Subjects

**DOI:** 10.3390/nu16040525

**Published:** 2024-02-14

**Authors:** Elisabetta Murru, Claudia Manca, Gianfranca Carta, Michele Ruggiu, Roberto Solinas, Roberta Montisci, Leanne Hodson, David Dearlove, Maria Pina Mollica, Filippo Tocco, Sebastiano Banni

**Affiliations:** 1 Department of Biomedical Sciences, University of Cagliari, 09042 Monserrato, Italy; m.elisabetta.murru@gmail.com (E.M.); claumanca@unica.it (C.M.); giancarta@unica.it (G.C.); 2 Clinical Cardiology and Sport Medicine, Department of Medical Science and Public Health, University of Cagliari, 09042 Monserrato, Italy; ruggiu.michele@gmail.com (M.R.); solirob@tiscali.it (R.S.); rmontisci@unica.it (R.M.); filippo.tocco@tiscali.it (F.T.); 3 Oxford Centre for Diabetes, Endocrinology and Metabolism, University of Oxford, Headington, Oxford OX3 7LE, UK; leanne.hodson@ocdem.ox.ac.uk (L.H.); david.dearlove@ocdem.ox.ac.uk (D.D.); 4 National Institute for Health Research Oxford Biomedical Research Centre, Oxford University Hospital Trusts, Oxford OX4 2PG, UK; 5 Department of Biology, University of Naples Federico II, 80126 Naples, Italy; mariapia.mollica@unina.it

**Keywords:** metabolic flexibility, indirect calorimetry, peroxisome proliferator-activated receptor α (PPARα), endocannabinoid system, *N*-oleoylethanolamine (OEA), 2-arachidonoylglycerol (2-AG)

## Abstract

We propose a novel method for assessing metabolic flexibility (MF) through indirect calorimetry. A total of twenty healthy volunteers (10 females; 10 males) aged 45–65 were categorized into a Low-Intensity activity group (LI, 0–1 session of 1 h per week) and a High-Intensity activity group (HI, 5–6 sessions of 2 h per week). Volunteers underwent a stepwise exercise test on a cycle ergometer, connected to a calorimeter, to examine respiratory gas exchange to evaluate peak fatty acid Oxidation (PFO) and peak carbohydrate oxidation (PCO). Circulating peroxisome proliferator-activated receptor α (PPARα) biomarkers, docosahexaenoic acid/eicosapentaenoic acid (DHA/EPA) ratio and *N*-oleoylethanolamine (OEA), and the endocannabinoid- 2-arachidonoylglycerol (2-AG), were evaluated. We developed two MF parameters: the MF index (MFI), calculated by the product of PFO normalized per kg of fat-free mass (FFM) and the percentage of VO_2max_ at PFO, and the peak energy substrates’ oxidation (PESO), computed by summing the kilocalories from the PFO and PCO, normalized per kg FFM. The MFI and PESO were significantly different between the HI and LI groups, showing strong correlations with the circulating bioactive substances. Higher DHA/EPA ratio (*p* ≤ 0.05) and OEA (*p* ≤ 0.01), but lower 2-AG levels (*p* ≤ 0.01) were found in the HI group. These new parameters successfully established a functional link between MF and the balance of PPARα/endocannabinoid systems.

## 1. Introduction

The term metabolic flexibility (MF) refers to the ability of an organism’s metabolism to maintain energy homeostasis by providing fuel availability under different metabolic/physiological conditions including periodic fasting, meal composition, physical activity, and environmental factors [1]. Recently, MF has expanded to include different metabolic conditions in tissues and more generally, to explain physiological adaptability [1]. MF could be considered a system of cooperating components to manage energy reserves and requirements in health and disease [2]. Chronic degenerative diseases, such as insulin resistance, type 2 diabetes, and obesity, are characterized by metabolic inflexibility. It has been shown that impaired insulin-stimulated glucose metabolism and reduced lipid catabolism lead to lower lipid depot into the subcutaneous adipose tissue and higher lipid depot in visceral fat, liver and skeletal muscle [3,4]. This metabolic imbalance fosters the development of lipotoxicity, thereby contributing to mitochondrial “indecision” [5] correlated with aging-associated physiological regression. This decline includes a progressive reduction in muscle mass and an increase in visceral adipose tissue [6,7,8]. MF is negatively correlated with aging [9] and its control may provide cues to delay the onset of age-related diseases. A recent study in women over 60 confirmed an impaired switching of substrate oxidation in response to exercise, in comparison to young women who are active cyclists, limiting their power output and affecting exercise capacity [10]. However, this work showed that an age-related decrease in muscle power, which typically refers to the ability to exert a maximal amount of force in the shortest possible time [11], had a key role above age per se, in the onset of metabolic inflexibility [10]. To account, given that the aging process is associated to a drop in fibers type II, expressed as the percentage of each type of fiber regarding the total [12], the glycolytic pathway might have been punished, resulting in lower MF. These observations confirm the link between muscle power, mitochondrial health, and the maintenance of MF. Despite the deterioration with age, muscle power is very sensitive to training [13] and may enhance MF. Physical exercise is a physiological condition requiring MF to balance fuel availability with the metabolic adjustments to meet considerable increases in skeletal muscle energy demands. Carbohydrates (CH) and fatty acids (FA) are substrates largely responsible for energy supply during exercise [14,15,16], and their contribution is influenced by the intensity and duration of exercise and training status, sex, and nutritional input [17]. Studies have indicated the existence of large inter-individual differences in the ability to oxidize FA during exercise [18,19]. Peak FA oxidation (PFO), which represents the maximum FA oxidation (FAO) level, varies between trained and untrained men and women [20]. Endurance-trained athletes exhibit enhanced oxidative capacity within muscle tissues, which facilitates FAO. This metabolic shift helps conserve intramuscular glycogen stores and reduces glucose oxidation [21]. In individuals with a highly glycolytic muscle fiber phenotype, MF may be somewhat limited. While they are very efficient at utilizing glucose as an energy source during high-intensity exercise, they may be less efficient at metabolizing fat during lower-intensity activities. However, muscle fiber type is not the sole determinant of MF. Other factors such as diet, physical fitness, body composition, and insulin sensitivity can influence how efficiently an individual can switch between different energy substrates [17,22].

Additionally, the accumulation of lactic acid (LA) could potentially influence metabolic adaptability. This was suggested in a study conducted by San-Millan and Brooks [23], which postulated that analyzing LA concentrations and FAO rates during physical exercise may offer an indirect approach to assess the MF and oxidative capacity among individuals exhibiting extensive variability in their metabolic capabilities.

Evaluation of MF necessitates consideration of receptor-mediated metabolic mechanisms involved in energy homeostasis, such as the endocannabinoid and peroxisome proliferator-activated receptor (PPAR) systems. The endocannabinoid system (ECS) comprises two G-protein-coupled receptors, specifically cannabinoid receptors type-1 and -2 (CB1 and CB2), their predominant endogenous ligands, namely *N*-arachidonoylethanolamine or anandamide (AEA) and 2-arachidonoylglycerol (2-AG), and enzymes that modulate their tissue levels. Apart from regulating central and peripheral nervous system functions, the ECS significantly influences various aspects of mammalian physiology including energy intake, processing and storage, immune response, and its dysregulation have been associated with obesity and metabolic syndrome pathogenesis [24]. Physical activity is recognized as a significant modulator of the ECS, mediating some of the systemic impacts of exercise [25]. Exercise influences the expression of receptors and enzymes involved in the synthesis and degradation of endocannabinoids, potentially affecting the anti-inflammatory effects of physical activity. Positive correlations have been observed between the endocannabinoid 2-AG levels in blood and cardiometabolic risk factors including body mass index (BMI), waist girth, and fasting plasma triglyceride and insulin levels in both genders [26,27].

PPARα is pivotal to MF, particularly in promoting FAO [28]. This nuclear receptor acts as a transcription factor, primarily regulating genes associated with lipid metabolism. In conditions such as fasting or prolonged exercise, where the energy source shifts from glucose to fats, PPARα activation enhances the transcription of genes involved in FA uptake, binding, and β-oxidation, promoting FA metabolism [29,30]. PPARα promotes MF by facilitating an efficient switch of energy sources based on availability and demand, thereby enhancing FAO when required. It underscores the vital role PPARα plays in maintaining energy homeostasis under different physiological conditions. The two endocannabinoid-like molecules, *N*-oleoylethanolamine (OEA) and *N*-palmitoylethanolamine (PEA) are endogenous lipids that activate PPARα [31].

Considering the many factors influencing MF, our present study seeks to evaluate MF using a novel method, analyzing the metabolic responses to exercise across an incremental test of intensities with a cyclo-ergometer via indirect calorimetry. We examined the correlation among PFO, peak CH oxidation (PCO), and maximum oxygen consumption (VO_2max_) during stepped-intensity exercise until voluntary fatigue in middle-aged (from 45 to 65 years), normal-weight individuals with varying oxidative capacities. This life stage is critical for the increase in the incidence of risk factors for many pathological conditions like metabolic syndrome, type 2 diabetes mellitus, and cardiovascular disease [32] that represent an economic burden for public health.

We propose two new quantifiable parameters: a metabolic flexibility index (MFI), calculated by the product of PFO, normalized per kg of fat-free mass (FFM), and %VO_2max_ at PFO; and peak of energy substrate’ oxidation (PESO), obtained by the sum of kcal PFO and PCO, normalized per kg of FFM. Furthermore, we intend to evaluate different PPARα and endocannabinoid-related circulating metabolic parameters and their potential changes in relation to MFI and PESO.

## 2. Materials and Methods

The present study was conducted according to the Declaration of Helsinki and all procedures involving individuals were approved by the ethical committee of A.O.U.CA (Azienda Ospedaliera Universitaria di Cagliari, approval code: prot. PG/2021/16466, approval date 27 October 2021). The trial was registered at ClinicalTrials.gov (prot. no. NCT06012227). Written informed consent was obtained from all subjects.

### 2.1. Experimental Design

A total of twenty healthy volunteers, ranging from sedentary individuals to those engaged in professional physical activity (10 males and 10 females), aged 45 to 65 years, were recruited from the Coronary Intensive Care Unit (UTIC) of the University Hospital of Cagliari. This age range was chosen due to its susceptibility to an elevated incidence of risk factors associated with various metabolic pathological conditions. The subjects’ health status was assessed through a questionnaire. Exclusion criteria included subjects with BMI < 19 and >28, and waist–hip ratio < 0.91 for men and <0.79 for women, with systemic diseases such as diabetes mellitus, liver disorders, neoplasms, collagen vascular diseases, plasma total cholesterol or triglycerides higher than 250 mg/dL, and a history of recent acute illness. The subjects were classified to one of two groups according to the degree of the usual training: a Low-Intensity (LI) activity group (0–1 session of moderate workout METs < 6.0, 1 h each/session) (F = 5; M = 4) and a High-Intensity (HI) activity group (5–6 sessions of intense workout, METs > 6.0, 2 h each/session), (F = 5; M = 6) expecting a different MF between these two groups.

Following an overnight fast of at least 12 h, volunteers arrived at the clinic to undergo the experimental trial. A maximal cardiopulmonary exercise test (CPET) was performed by each participant during a cyclo-ergometer (Ergoline Bosch 500, Berlin, Germany) incremental test. Oxygen consumption (VO_2_) and carbon dioxide production (VCO_2_) were collected by a breath-by-breath analysis (Ergostik, Geratherm Medical AG, Geschwenda, Germany) and heart rate (HR) was continuously recorded by a 12-lead electrocardiogram (Mortara XScribe stress test, Milwaukee, WI, USA) under the supervision of a cardiologist. Blood pressure measurements were carried out at rest, during exercise, and at recovery.

After a 3 min resting period in an upright position seated on the cycle ergometer to establish baseline data, the subjects started pedalling at 60 rpm with the workload progressively increased by 15 or 30 watts every 5 min (to allow the achievement of a metabolic steady state) until volitional exhaustion, indicated by the subject’s inability to maintain a pedalling rate of at least 50 rpm. The workload achievable by each individual was estimated by taking into account factors such as body mass and training status, ensuring a personalized and appropriate level of exercise intensity.

Volitional exhaustion was confirmed in all subjects by reaching the heart rate corresponding to their age and theoretical VO_2max_. This makes it unlikely that they terminated the exercise prematurely due to psychological factors such as anxiety about breathlessness.

Venous blood samples were taken from the antecubital vein prior to the cyclo-ergometer test to determine differences in circulating lipid parameters between the two groups. For the assessment of glycaemia (Gly) and circulating LA concentrations, a drop of venous blood before exercise, at the end of each step, and at the 3rd minutes of the recovery period were utilized.

### 2.2. Lactic Acid (LA) Turn-Points

For the identification of the two LA thresholds, we adopted the Individual Anaerobic Threshold (IAT) method [33], since LA values vary according to individual characteristics (ratio of slow and fast muscle fibers). Thus, we have not considered the absolute values of 2 mMol and 4 mMol as indices of the first and second threshold, but as the trend of the curve obtained from the LA/power graph. The first inflection corresponds to the first threshold and marks the beginning of the phase in which the LA production is compensated by the removal. The second inflection identifies the second threshold, i.e., the beginning of the phase in which LA production is not compensated for removal with consequent accumulation.

### 2.3. Maximal Oxygen Consumption

During the tests, the gas analyzer provided averaged data every 5–7 s. The acquired data were collected and analyzed by averaging the amount of the last 1 min of each step. The results obtained were then interfaced with the relative power held by the athlete and reported on Cartesian graphs, which showed the workload on the abscissa and the oxygen consumption on ordered axis. The calculated VO_2max_ was considered valid if it met the achievement of these two criteria: (1) respiratory exchange ratio > 1.10; (2) and a HR ± 10 beats/min (bpm) of predicted maximum HR calculated as 220 minus the age [34].

### 2.4. Fatty Acid (FA) and Carbohydrate (CH) Oxidation Rate Analyses

The percentage of oxidized CH and FA can be assessed by measuring the ratio between the metabolic production of CO_2_ and the uptake of O_2_ with the Respiratory Exchange Ratio (RER), using indirect calorimetry: a ratio of around 0.7 means greater FAO whilst a ratio up to 1 occurs when CH metabolism is dominant [35]. For the measurement of total FAO and CH Oxidation (CHO), stoichiometric equations were applied according to the methodology described by Frayn [36]; FAO (g/min) was calculated by 1.67 VO_2_ (L/min) − 1.67 VCO_2_ (L/min) and CHO was calculated by 4.55 VCO_2_ (L/min) − 3.21 VO_2_ (L/min).

### 2.5. Blood Sampling

Venous blood samples taken from the antecubital vein were collected into anticoagulant-coated vacutainers (K3 EDTA) and centrifuged at 2000× *g* for 15 min at room temperature. The plasma was separated from the red blood cells and stored at −80 °C until the quantification of total lipids and fatty acids profiling.

### 2.6. Lipid Analyses

Lipids were extracted from human plasma using a modified Folch method [37]. Total lipid quantification was performed as described by Chiang [38].

An aliquot of the plasma lipid extract was mildly saponified and unsaturated FA were analyzed by HPLC using an Agilent 1100 HPLC system (Agilent Technologies, Palo Alto, CA, USA), as previously described [39]. Saturated FA, after derivatization, were measured as FA methyl esters using gas chromatography (GC; Agilent Model 6890, Agilent Technologies), as previously described [40].

Another aliquot of the plasma lipid fraction was used to analyze *N*-acylethanolamine (NAE) and was carried out by an Agilent 1260 UHPLC system (Agilent, Palo Alto, CA, USA) equipped with a mass spectrometry Agilent Technologies QQQ triple quadrupole 6420 with ESI source, using positive mode (ESI+). A Poroshell 120 EC-C-18 column (Agilent, Palo Alto, CA, USA) with 2.7 μm particle size and 3 × 100 mm was used with a mobile phase of CH_3_OH/H_2_O/CHOOH (90/10/0.1, *v*/*v*/*v*) at a flow rate of 0.5 mL/min. N_2_ was used as a nebulizing gas with a pressure of 50 psig, a drying gas temperature of 300 °C, a flow of 11 L/min, and 4000 V capillary voltage. For each standard, the precursor ion [M + H]^+^ was determined during a full scan (SCAN) in MS, and subsequently, the obtained product ion (PI) was monitored for each transition in MRM mode in MS/MS. Parameters of source, such as cone voltage or fragmentor (CV) and collision energy (CE), have been optimized for each MRM transition [40].

Data were acquired by the MassHunter workstation acquisition software (version B.08.02) and analyzed with the MassHunter software for qualitative analysis (version B.08.00 SP1) and quantitative analysis (version B.09.00).

### 2.7. Statistical Analysis

Data are expressed as mean ± Standard Deviation (SD). Data were analyzed using the GraphPad Prism 6.01 software (La Jolla, CA, USA). Since data were normally distributed (Shapiro–Wilk normality test), the HI and LI data were evaluated using unpaired Student’s *t*-test (two-tailed) with a 95% CI. Correlations among the key markers in the study of the MF were assessed using the nonparametric Spearman correlation (two-tailed) coefficient with a 95% CI. Statistical significances were indicated as follows: * *p* ≤ 0.05; ** *p* ≤ 0.01; *** *p* ≤ 0.001.

## 3. Results

### 3.1. Anthropometric and Performance Characteristics of Subjects

The present study aimed to assess the response to physical exercise in individuals classified as either HI or LI. Body weight and BMI were not significantly different between the two groups, but the percentage of fat mass (FM) was found to be higher by 40% in the LI group compared to the HI group (*p* ≤ 0.01, Eta^2^ 0.323) (Table 1).

Table 2 and Table 3 display the resting and post-exercise parameters.

Resting Gly and total lipids did not differ significantly between the HI and LI groups. Conversely, post-exercise Gly at 1 min of recovery, as a percentage of basal levels, was significantly increased by 15% in the HI group (*p* ≤ 0.01, Eta^2^ 0.297), while in the LI group it was decreased by 14% (Table 2 and Table 3).

Resting HR was significantly higher by 23% (*p* ≤ 0.01, Eta^2^ 0.367) in the LI group compared to the HI group. At VO_2max_, HR was 95% in the LI group and 130% in the HI group (*p* ≤ 0.05, Eta^2^ 0.309), relative to resting values. The HI group displayed a better exercise performance, reaching a higher maximum workload (WL_max_) at muscle exhaustion compared to the LI group. VO_2max_ was significantly (*p* ≤ 0.001, Eta^2^ 0.453) higher in the HI group than in the LI group. Blood LA in resting condition was significantly lower (*p* ≤ 0.05, Eta^2^ 0.304) in the LI group compared with the HI group (Table 2 and Table 3).

**Figure 1:**
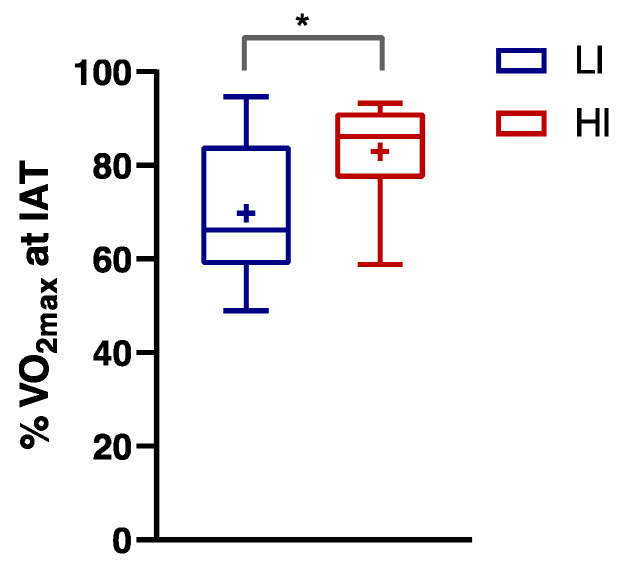
% VO_2max_ at the Individual Anaerobic Threshold (IAT) observed in Low-Intensity (LI, n = 9; ♀ = 5, ♂ = 4) and High-Intensity activity (HI, n = 11; ♀ = 5, ♂ = 6) subjects. Data were evaluated using unpaired Student’s *t*-test (two-tailed) with a 95% CI. Values are presented as box and whisker (higher and lower values); the symbol + represents the mean value. Statistical significances were * *p* ≤ 0.05.

By evaluating the %VO_2max_ at IAT, we found that the second LA turn point was reached at 70% VO_2max_ in LI subjects and 83% in HI subjects (Figure 1; *p* ≤ 0.05, Eta^2^ 0.306).

In the resting condition, RER did not show a significant difference between the two groups. However, at VO_2max_ it was significantly higher by 8% (*p* ≤ 0.01, Eta^2^ 0.347) in LI subjects compared to HI subjects. At VO_2max_, both groups exhibited RER values higher than one, indicating a respiratory compensation for metabolic acidosis (Table 2 and Table 3).

### 3.2. Numerically Measurable Parameters Indicative of Metabolic Flexibility (MF): MFI and PESO

**Figure 2:**
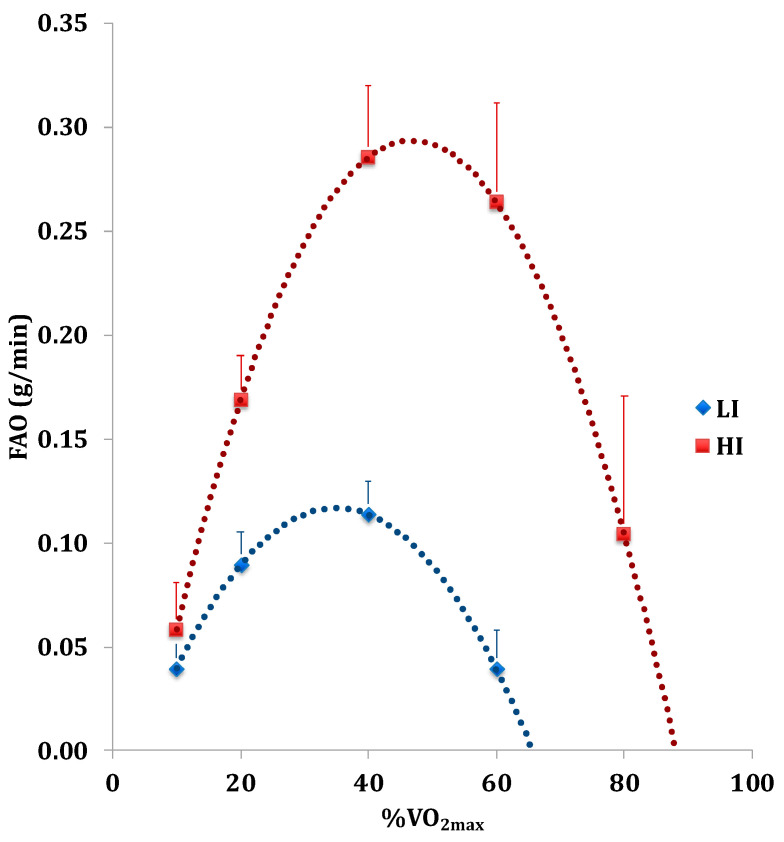
Fatty acid oxidation (FAO) rate in relation to %VO_2max_ during the incremental test in Low-Intensity activity (LI, n = 9; ♀ = 5, ♂ = 4) and High-Intensity activity (HI, n = 11; ♀ = 5, ♂ = 6) subjects.

By estimating FAO data, we graphically represented the FAO rate in relation to the percentage of VO_2max_ during the incremental exercise test (Figure 2), through a polynomial regression where the given data curve is approximated to a polynomial of the second degree to identify the curvilinear relationship between independent and dependent variables.

This polynomial regression is sensitive to outliers that are not in the given range (95% confidence level) because the presence of one or two outliers can also critically affect the data performance. The value of the PFO and the corresponding %VO_2max_ value were calculated by the polynomial curve removing outliers, while PCO was measured at 100% of VO_2max_. Specifically, we accounted for CO_2_ excess due to metabolic acidosis by replacing VCO_2_ (CO_2_ production) values with corresponding VO_2_ (O_2_ consumption) values to maintain the VCO_2_/VO_2_ ratio at 1. Moreover, the polynomial regression curve helps mitigate gender differences in muscular power by considering the maximum individual %VO_2max_ value. The PFO and VO_2max_ at PFO are strictly influenced by individual metabolic conditions during physical exercise. In respect to the initial value, PFO increased 5-fold in the HI group and 3-fold in the LI group.

**Figure 3:**
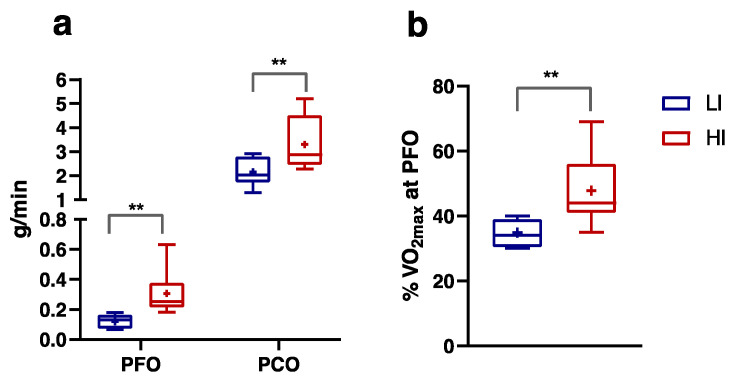
Peak fatty acid oxidation (PFO) and peak carbohydrates oxidation (PCO) expressed in g/min, (**a**) %VO_2max_ at PFO (**b**) during the incremental test in Low-Intensity (LI, n = 9; ♀ = 5, ♂ = 4) and High-Intensity (HI, n = 11; ♀ = 5, ♂ = 6) subjects. Data were evaluated using unpaired Student’s *t*-test (two-tailed) with a 95% CI. Values are presented as box and whisker (higher and lower values); the symbol + represents the mean value. Statistical significances were ** *p* ≤ 0.01.

Moreover, in LI subjects PFO and PCO were significantly reduced by 60% and 35%, respectively, compared to the HI group (Figure 3a). The %VO_2max_ at PFO was found to be 35 and 48 in LI and HI, respectively (Figure 3b).

**Figure 4:**
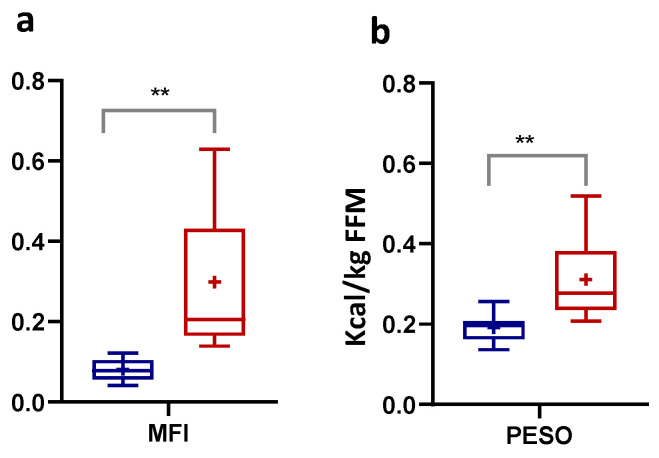
Metabolic flexibility index (MFI) calculated by (%VO_2max_ at PFO) X PFO, expressed in g/min/Kg FFM) (**a**), and peak energy substrate oxidation (PESO) index calculated by [(PFO + PCO, expressed in kcal/kg FFM] (**b**), during the incremental test in Low-Intensity activity (LI, n = 9; ♀ = 5, ♂ = 4) and High-Intensity activity (HI, n = 11; ♀ = 5, ♂ = 6) subjects. Data were evaluated using unpaired Student’s *t*-test (two-tailed) with a 95% CI. Values are presented as box and whisker (higher and lower values); the symbol + represents the mean value. Statistical significances were ** *p* ≤ 0.01.

We proposed an MFI calculated by the product between %VO_2max_ at PFO and PFO, normalized per kg of FFM to mitigate the impact of individual variability in body composition. A MFI could be considered to represent a unifying numerically measurable parameter for MF, displaying the ability to use FA as an energy substrate during incremental exercise. On the other hand, by evaluating the combination of PFO and PCO levels, we calculated the PESO, expressed in kilocalories (kcal) and obtained by the sum between PFO and PCO, normalized per kg of FFM. The average MFI value was higher in the HI group than the LI group, in which it was significantly reduced by 73% (Figure 4a, *p* ≤ 0.01, Eta^2^ 0.425), thus denoting a supposed metabolic inflexibility. In the LI group, the PESO value was significantly lower by 39% compared to the HI subjects, indicating a lower energy substrates disposal (Figure 4b, *p* ≤ 0.01, Eta^2^ 0.384).

### 3.3. Plasma FA, Endocannabinoids and Related NAE Profiles

**Figure 5:**
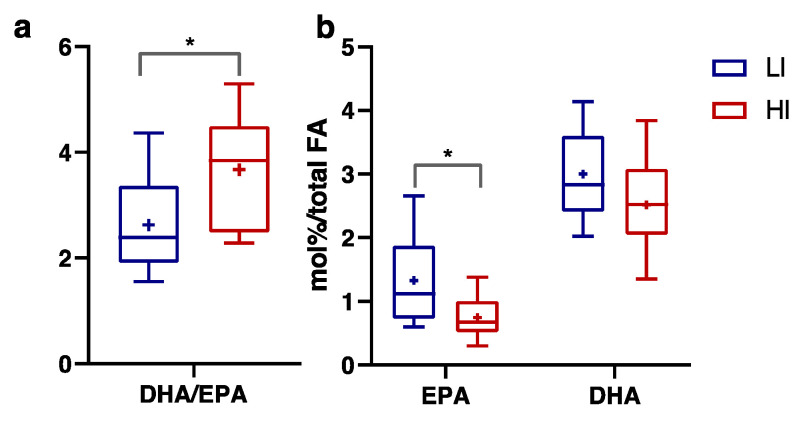
Docosahexaenoic acid/eicosapentaenoic acid (DHA/EPA) ratio (**a**), EPA levels expressed in mol% on total fatty acids and DHA levels expressed in mol% on total fatty acids (**b**) measured in Low-Intensity activity (LI, n = 9; ♀ = 5, ♂ = 4) and High-Intensity activity (HI, n = 11; ♀ = 5, ♂ = 6) subjects. Data were evaluated using unpaired Student’s *t*-test (two-tailed) with a 95% CI. Values are presented as box and whisker (higher and lower values); the symbol + represents the mean value. Statistical significances were * *p* ≤ 0.05.

Statistically significant changes in the circulating FA profile were limited to a higher docosahexaenoic acid (DHA) to eicosapentaenoic acid (EPA) ratio, considered a peroxisomal β-oxidation index [41], in the HI group compared to the LI group (Figure 5a, *p* ≤ 0.05, Eta^2^ 0.245). We observed lower EPA levels by 45% in the HI group (Figure 5b, *p* ≤ 0.05, Eta^2^ 0.247), while DHA did not differ between groups, suggesting that differences in the DHA/EPA ratio did not depend on their dietary intake, since they are both abundantly present in n-3 highly polyunsaturated fatty acid dietary sources [42] and thereby when they are from a dietary source, they may both increase proportionally.

**Figure 6:**
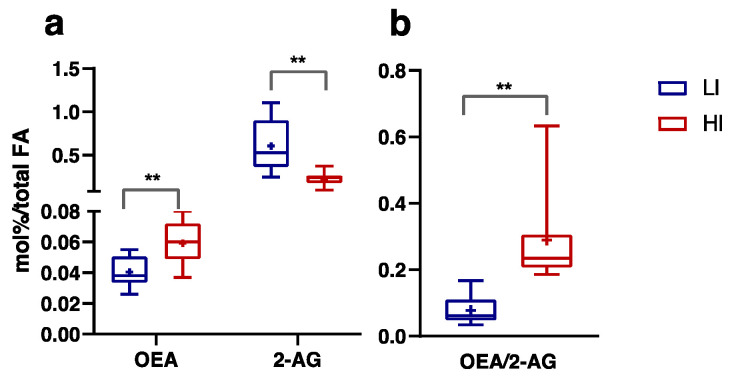
*N*-Oleoylethanolamine (OEA) levels and 2-arachidonoylglycerol (2-AG) levels, expressed in mol% on total fatty acids (**a**), and OEA/2-AG ratio (**b**) measured in Low-Intensity activity (LI, n = 9; ♀ = 5, ♂ = 4) and High-Intensity activity (HI, n = 11; ♀ = 5, ♂ = 6) subjects. Data were evaluated using unpaired Student’s *t*-test (Two-tailed) with a 95% CI. Values are presented as box and whisker (higher and lower values); the symbol + represents the mean value. Statistical significances were ** *p* ≤ 0.01.

Analysis of plasma endocannabinoids and related NAE showed that OEA levels were significantly higher in the HI group than the LI group (*p* ≤ 0.01, Eta^2^ 0.406). Conversely, 2-AG was significantly lower the HI group than the LI group (*p* ≤ 0.01, Eta^2^ 0.510) (Figure 6a). Consequently, the OEA/2-AG ratio was significantly higher in the HI group, with respect to the LI group (Figure 6b, *p* ≤ 0.01, Eta^2^ 0.482).

### 3.4. Correlations among the Key Markers of the Metabolic Flexibility (MF)

Table 4 presents the Spearman correlations among various parameters. The MFI and PESO exhibited a strong positive correlation (r = 0.73; *p* ≤ 0.001). Neither of these indices showed any correlation with age, whereas age was positively correlated with weight and BMI.

The metabolic parameters (WL_max_, VO_2max_, VO_2max_ at PFO, PFO, PCO), and consequently the MFI and PESO, demonstrated a negative correlation with percentage of body fat (% FM), but no correlation with BMI.

The Gly at 1 min of recovery, expressed as a percentage of basal levels, showed a positive correlation with both metabolic parameters (MFI and PESO) and circulating parameters (DHA/EPA ratio and OEA). The DHA/EPA ratio displayed a negative correlation with age, weight, BMI, %FM, and circulating 2-AG levels. In contrast, it exhibited positive correlations with %VO_2max_, PCO, MFI, PESO, and OEA levels.

OEA demonstrated a negative correlation with BMI and positive correlations with MFI, PESO, and VO_2max_. On the other hand, 2-AG exhibited a positive correlation with BMI.

The OEA/2-AG ratio, which serves as a potential index of the balance between endogenous PPARα and ECS activation, showed a negative correlation with weight and BMI, and a positive correlation with the DHA/EPA ratio.

## 4. Discussion

In the literature, two main approaches have been described for evaluating MF, each involving the monitoring of changes in substrate oxidation under different physiological conditions. The first is a nutritional approach, where the focus is on observing substrate oxidation in response to varying dietary patterns, specifically by altering the carbohydrate-to-fat ratio in the diet [43,44]. The second approach involves assessing substrate oxidation during a graded exercise test on a cycle ergometer [23]. In our study, we chose to utilize the exercise test approach. This decision was partly due to our intention to also investigate changes in PPARα and endocannabinoid biomarkers. These biomarkers are significantly influenced by dietary fat content [45], and a nutritional approach could potentially obscure the understanding of the role of PPARα and the endocannabinoid system in MF. San-Millan and Brooks [23] proposed the examination of LA levels and FAO rates during physical activity as an indirect method to evaluate MF. Nonetheless, this approach failed to incorporate a consideration of the extent of capacity to oxidize FA as an energy substrate during exercise, as well as the maximum capacity for utilizing both major energy substrates, namely glucose and FA.

In this study we introduce two novel numerically quantifiable parameters pertinent to efficient metabolic capacity for performing physical activity and thereby optimizing energy substrate usage. The first parameter, designated as the MFI, is calculated by multiplying the PFO rate, normalized per kg of FFM, by the percentage of VO_2max_ at which PFO occurs. This intersection point marks the threshold at which PFO begins to decrease, and CHO steeply increases, contributing to the concept of MF. Therefore, MFI considers the PFO relative to the expanding capacity to metabolize FA as substrates in a stepwise increment in %VO_2max_. The second parameter, known as PESO, is derived by summing the kcal from PFO and PCO, normalized per kg FFM. This reflects the maximum capacity to oxidize both energy substrates.

These parameters yield distinct sets of information and can be applied independently. The MFI specifically addresses the capacity for fat utilization as an energy substrate, emphasizing the duration and efficiency of fat oxidation. In contrast, PESO, inclusive of the PCO component, accounts for the maximal energy expenditure derived from both lipid and glucose substrates. Thus, these two parameters reveal different aspects of MF: MFI illustrates the ability and endurance of fat utilization as an energy source, a key element of MF, while PESO, through PCO, also encompasses the availability and utilization of glucose, another critical facet of MF. Notably, PESO demonstrates a stronger correlation with blood glucose levels during recovery phases compared to MFI, highlighting its relevance in glucose metabolism assessment.

Hence, collectively, these two parameters encapsulate the efficiency of optimizing the utilization of energy substrates in alignment with metabolic demand.

These parameters may be used to assess the success of personalized strategies to improve and delay physiological decline, particularly with regard to reduced muscle mass and impaired adipose tissue distribution. In fact, we suggest that MF encompasses not only the ability to optimally use energy substrates but also the ability to adequately store them, thus allowing the conservation of metabolic homeostasis following acute challenges, favouring cross-talk between fat and muscle mass, impeding dynapenia [46] and favouring an optimal body composition.

The storage of glucose as glycogen plays a crucial role in utilizing glucose as an energy substrate. Glycogen storage in muscles is well known for its significance in endurance training [47]. However, during fasting conditions, optimal storage of glycogen in the liver also plays a vital role in maintaining glucose homeostasis. An increased capacity for liver glycogen storage may lead to a more efficient hepatic ability to maintain glucose homeostasis during exercise stress, involving a combination of stimulated hepatic glucose release and activated peripheral glucose uptake.

The observed 15% increase in Gly in the HI group and the 14% decrease in the LI group during the recovery phase may be attributed to the availability of liver glycogen for glucose release and utilization by the muscles during exercise. As the demand for glucose by the muscles reduces during the recovery phase, there may be an increment of Gly in the HI group.

It is likely that the activation of the sympathetic nervous system during exercise stimulates glycogenolysis in both muscles and the liver, as well as lipolysis in adipose tissue, as an initial endocrine response to exercise stress. This response has been shown to be accompanied by an increase in plasma cortisol and epinephrine levels, which may contribute to enhancing glucose availability during exercise [48,49]. In our specific experimental conditions, indeed, we noted a rapid response of liver glycogenolysis.

This prompt liver glycogenolysis response supports the notion that it may contribute to the MF. Furthermore, our findings revealed a strong and significant correlation between MFI or PESO with the percentage of Gly at 1 min into the recovery phase after exercise, compared to the baseline levels.

It has been reported that a decreased mitochondrial function affects LA clearance capacity, and exerts profound effects on energy substrate oxidation, as FA and CH [23].

We confirmed, as previously shown [50], that resting circulating LA is lower in the LI individuals compared with the HI subjects. Seemingly, at VO_2max_, blood LA level was increased, but not significantly, in HI versus LI individuals. It is plausible this occurred because of the higher variability, due to a greater difference in the maximum workload, between the HI and LI groups. We showed that the percentage VO_2max_ at the IAT, in which the removal does not compensate LA production, was lower in LI subjects than HI subjects (70% and 83%, respectively). This may be explained by a better aerobic capacity in HI, but also by a more efficient trans-sarcolemmal LA transport [51] in trained subjects [52]. The higher aerobic capacity in HI may be ascribed to muscular differences such as mitochondria volume, muscle dense capillary, enzymatic activities, and a high percentage of type I fibers [53], which may enhance LA uptake and oxidation, permitting to reach a higher percentage VO_2max_ at IAT in trained subjects in comparison with untrained subjects. On the other hand, in LI subjects, we observed a higher RER at 100% VO_2max_ with respect to the HI group, caused by an increase in the LA buffering [54].

We also investigated the potential association between the MFI and PESO with circulating lipid metabolites known to be involved in MF. Our findings revealed strong correlations between the MFI, PESO, and various PPARα-related circulating lipid metabolic parameters. These results suggest a significant role of PPARα in promoting MF, consistent with previous pre-clinical studies [55].

Specifically, the MFI and PESO were positively correlated with the DHA/EPA ratio, which serves as a marker of increased peroxisomal β-oxidation, regulated by PPARα [41]. Additionally, they were positively correlated with EC-related mediators, such as OEA, an avid PPARα ligand [28]. Interestingly, the MFI exhibited a negative correlation with the endocannabinoid 2-AG, a ligand of CB1 receptors [56], which is associated with promoting palatable food intake, appetite motivation, and fat accumulation, thereby impairing MF. 2-AG has been found to increase with obesity and represents a marker of an overactive ECS [26].

It is important to note that the strong correlation between the MFI, PESO, and plasmatic lipid biomarkers associated with PPARα and ECS activities does not necessarily imply causality.

Albeit this study provides valuable insights into the assessment of MF and its association with circulating biomarkers of PPARα and ECS activity, it is important to acknowledge certain limitations that necessitate a cautious interpretation of the results. The primary limitation is the relatively small sample size. This limitation was primarily due to the preliminary nature of our research, which involved extensive testing and optimization of our analytical procedures and data analysis methods.

Given the exploratory and novel aspects of our study, we chose to focus on a smaller, more manageable cohort. This approach allowed us to maintain a high degree of control and precision in our experimental setup and data collection processes.

## 5. Conclusions

Higher levels of circulating OEA and DHA/EPA ratio, along with lower levels of circulating 2-AG in individuals with HI activity, support the hypothesis that MF is promoted by enhanced PPARα activity and reduced ECS activation. This is further supported by a 70% lower OEA/2-AG ratio in LI subjects, suggesting that the balance between PPARα and ECS may play a role in regulating MF.

Future research with expanded cohorts is essential to fully understand and validate the implications of our findings and investigate whether and how the MFI and PESO can be modified through nutritional means aimed at altering the PPARα/ECS balance [26,55,57,58,59,60,61]. Additionally, it would be valuable to assess whether specific personalized training in individuals with low MFI and PESO values can modulate the PPARα/ECS balance.

Establishing a functional link between MF and the PPARα/ECS balance will highlight the importance of using the proposed MF parameters to tailor personalized nutritional and physical activity interventions for preserving MF in middle-aged individuals.

## Figures and Tables

**Table 1:** Anthropometric characteristics in LI (n = 9; ♀ = 5, ♂ = 4) and HI (n = 11; ♀ = 5, ♂ = 6) subjects.

Anthropometric Measures	LI (n = 9)	HI (n = 11)	*p*-Value	Eta^2^
Sex	♀ = 5, ♂ = 4	♀ F = 5, ♂ M = 6		
Age (years)	52.00 ± 8.03	48.73 ± 6.57	0.329	0.053
Height (m)	1.66 ± 0.09	1.69 ± 0.07	0.462	0.030
Body Weight (kg)	69.22 ± 13.41	64.27 ± 9.00	0.337	0.051
BMI (kg/m^2^)	25.02 ± 3.78	22.45 ± 1.64	0.056	0.188
FM (%)	25.53 ± 6.41	18.05 ± 4.66	**0.007**	0.323
FFM (%)	75.64 ± 4.73	81.98 ± 4.16	**0.005**	0.323

LI, low-intensity activity; HI, high-intensity activity; BMI, body mass index; FM, fat mass; FFM, free fat mass. Data were evaluated using unpaired Student’s *t*-test (two-tailed) with a 95% CI. Values were statistically significant with *p* ≤ 0.05 (in bold when *p*-value is significant).

**Table 2:** Resting parameters in LI (n = 9; ♀ = 5, ♂ = 4) and HI (n = 11; ♀ = 5, ♂ = 6) subjects.

Resting Parameters	LI (n = 9)	HI (n = 11)	*p*-Value	Eta^2^
Gly (mg/dL)	108.71 ± 8.04	102.70 ± 10.35	0.218	0.099
Total lipids (mg/mL)	4.99 ± 0.93	4.69 ± 0.68	0.419	0.037
SBP (mmHg)	118.89 ± 9.28	113.18 ± 14.19	0.314	0.056
DBP (mmHg)	76.67 ± 7.07	75.45 ± 8.20	0.731	0.007
HR (bpm)	88.00 ± 15.94	71.43 ± 5.47	**0.005**	0.367
LA (mmol/L)	1.59 ± 0.27	2.01 ± 0.38	**0.018**	0.304
RER (V_CO2_/V_O2_)	0.85 ± 0.11	0.78 ± 0.08	0.129	0.120

LI, low-intensity activity; HI, high-intensity activity; Gly, Glycaemia; SBP, Systolic blood pressure; DBP, Diastolic blood pressure; HR, heart rate; LA, lactic acid; RER, Respiratory Exchange Ratio. Data were evaluated using unpaired Student’s *t*-test (two-tailed) with a 95% CI. Values were statistically significant with *p* ≤ 0.05 (in bold when *p*-value is significant).

**Table 3:** Post-exercise characteristics in LI (n = 9; ♀ = 5, ♂ = 4) and HI (n = 11; ♀ = 5, ♂ = 6) subjects.

Post-Exercise Parameters	LI (n = 9)	HI (n = 11)	*p*-Value	Eta^2^
Maximum workload (watt)	123.89 ± 38.87	214.09 ± 67.41	**0.002**	0.412
VO_2max_ (ml/min/kg)	23.31 ± 4.43	38.38 ± 10.97	**0.001**	0.453
RER at VO_2max_	1.15 ± 0.08	1.06 ± 0.05	**0.007**	0.347
Gly at recovery (% of basal value)	93.96 ± 10.44	118.56 ± 24.15	**0.002**	0.297
HR at VO_2max_ (bpm)	168.11 ± 15.48	164.48 ± 15.35	0.606	0.015
HRR (%)	95.35 ± 32.40	130.99 ± 23.74	**0.011**	0.309
HRr (%)	6.43 ± 1.20	9.64 ± 2.59	**0.006**	0.408
LA at VO_2max_ (mmol/L)	6.13 ± 1.62	7.94 ± 2.82	0.107	0.138

LI, low-intensity activity; HI, high-intensity activity; WL_max_, maximum workload; VO_2max_, maximum oxygen consumption; RER, Respiratory Exchange Ratio; Gly, Glycaemia; HR, heart rate; HRR, heart rate increment versus resting levels; HRr, heart rate variation at 1 min of recovery versus maximum levels; LA, lactic acid. Data were evaluated using unpaired Student’s *t*-test (two-tailed) with a 95% CI. Values were statistically significant with *p* ≤ 0.05 (in bold when *p*-value is significant).

**Table 4:** Spearman correlation coefficients among the key markers in the study of the metabolic flexibility (MF). Statistical significance as follow: * *p* ≤ 0.05; ** *p* ≤ 0.01; *** *p* ≤ 0.001.

Parameters	Age	Weight	%FM	BMI	WL_max_	%VO_2max_	%VO_2 max_ at PFO	PFO	PCO	MFI	PESO	%Gly at Recovery	DHA/ EPA	OEA	2AG	OEA/ 2-AG
Age	1															
Weight	0.46 *	1														
%FM	0.19	0.03	1													
BMI	0.49 *	0.81 ***	0.42	1												
WL_max_	−0.21	0.15	−0.81 ***	−0.12	1											
%VO_2max_	−0.37	−0.15	−0.80 ***	−0.33	0.92 ***	1										
%VO_2 max_ at PFO	−0.09	−0.30	−0.63 **	−0.39	0.60 **	0.72 ***	1									
PFO	−0.22	−0.06	−0.79 ***	−0.27	0.85 ***	0.92 ***	0.80 ***	1								
PCO	−0.21	0.10	−0.65 **	−0.06	0.75 ***	0.75 ***	0.40	0.73 ***	1							
MFI	−0.25	−0.29	−0.71 ***	−0.42	0.71 ***	0.85 ***	0.92 ***	0.94 ***	0.60 **	1						
PESO	−0.41	−0.36	−0.60 **	−0.40	0.59 **	0.74 ***	0.57 **	0.72 ***	0.88 ***	0.73 ***	1					
%Gly at recovery	−0.43	−0.22	−0.37	−0.32	0.47 *	0.55 *	0.33	0.57 *	0.64 **	0.50 *	0.69 **	1				
DHA/EPA	−0.46 *	−0.58 **	−0.44 *	−0.53 *	0.31	0.56 **	0.39	0.40	0.48 *	0.48 *	0.70 **	0.49 *	1			
OEA	−0.29	−0.39	−0.42	−0.53 **	0.37	0.47 *	0.37	0.42	0.35	0.43	0.50 *	0.64 **	0.58 **	1		
2AG	0.09	0.45	0.27	0.53 *	−0.30	−0.34	−0.35	−0.32	−0.18	−0.36	−0.30	−0.25	−0.50 *	−0.39	1	
OEA/2-AG	−0.21	−0.50 *	−0.29	−0.54 *	0.21	0.31	0.31	0.23	0.13	0.28	0.31	0.35	0.72 ***	0.62 **	−0.69 **	1

FM, Fat Mass; BMI, Body Mass Index; WL_max_, maximum workload; PFO, Peak Fatty acid Oxidation; PCO, Peak Carbohydrate Oxidation; MFI, Metabolic Flexibility Index; PESO, Peak Energy Substrate’ Oxidation; Gly, glycaemia; EPA, eicosapentaenoic acid; DHA, docosahexaenoic acid; OEA, *N*-Oleoylethanolamine; 2-AG, 2-Arachidonoylglycerol.

## Data Availability

The data presented in this study are available on request from the corresponding author. The data are not publicly available in accordance with consent provided by participants on the use of confidential data.

## Abbreviations

Metabolic flexibility (MF), Carbohydrates (CH), fatty acids (FA), carbon dioxide (CO_2_), oxygen (O_2_), Respiratory Exchange Ratio (RER), Peak FA oxidation (PFO), lactic acid (LA), FA oxidation (FAO), peroxisome proliferator-activated receptor (PPAR), endocannabinoid system (ECS), cannabinoid receptors (CB), *N*-arachidonoylethanolamine (AEA), 2-arachidonoylglycerol (2-AG), *N*-oleoylethanolamine (OEA), *N*-palmitoylethanolamine (PEA), peak carbohydrate oxidation (PCO) and maximum oxygen consumption (VO_2max_), Metabolic Flexibility Index (MFI), body mass index (BMI), fat mass (FM), fat-free mass (FFM), Peak of Energy Substrate Oxidation (PESO), Low Intensity activity (LI), High Intensity activity (HI), cardiopulmonary exercise test (CPET), heart rate (HR), Individual Anaerobic Threshold (IAT), maximum workload (WLmax), Systolic blood pressure (SBP), Diastolic blood pressure (DBP), beat/min (bpm), *N*-acylethanolamines (NAE), glycaemia (Gly).
